# Quality of life in dysphagia and functional performance of cancer patients in palliative care

**DOI:** 10.1590/2317-1782/20242023266en

**Published:** 2024-09-09

**Authors:** Laressa Cardoso Barbosa, Leandro de Araújo Pernambuco, Hipólito Magalhães

**Affiliations:** 1 Programa de Pós-graduação em Fonoaudiologia, Universidade Federal do Rio Grande do Norte – UFRN - Natal (RN), Brasil.; 2 Departamento de Fonoaudiologia, Universidade Federal de Pernambuco – UFPE - Recife (PE), Brasil.; 3 Departamento de Fonoaudiologia, Universidade Federal do Rio Grande do Norte – UFRN - Natal (RN), Brasil.

**Keywords:** Deglutition Disorders, Quality of Life, Palliative Care, Medical Oncology, Neoplasms, Functional Status

## Abstract

**Purpose:**

To correlate the functional performance and impact of dysphagia on the quality of life of cancer patients in palliative care.

**Methods:**

This cross-sectional, quantitative study was conducted at the outpatient clinic and oncology ward of a university hospital. Inclusion criteria required patients to respond positively to the question: “Do you have difficulty or problems swallowing?”. Patients were excluded if they had been diagnosed with head and neck cancer, were unable to answer questionnaires due to actively dying status, were in a state of drowsiness, experienced extreme pain and systemic instability, or if data collection instruments were incomplete. Two instruments were used in their Brazilian Portuguese versions: the Palliative Performance Scale (PPS) and the M. D. Anderson Dysphagia Inventory (MDADI). The variables were analyzed using descriptive and inferential statistics, with Pearson's correlation used at a 5% significance level.

**Results:**

The sample consisted of 39 participants, with an average age of 65.3 years, of whom 24 (61.5%) were women. The most frequent neoplasm sites were the pancreas and stomach. The results of the PPS indicated that the average patient had reduced ambulation and inability to work, but maintained independence in self-care, with a complete level of swallowing and consciousness. The MDADI showed an average degree of limitation. Outpatients exhibited a moderate correlation between the MDADI result and the level of functionality according to the PPS.

**Conclusion:**

Cancer patients at the palliative care outpatient clinic demonstrated a correlation between functional performance and the impact of dysphagia on quality of life.

## INTRODUCTION

Cancer is one of the major health problems that affects all populations, observed more prevalently in middle and low-income countries due to limited resources invested in health, whether in prevention or treatment^(1)^. The most prevalent types include lung cancer (11.6% of all cases), followed by breast cancer in women (11.6%) and colorectal cancer (10.2%). The WHO estimated that 18.1 million people worldwide had some form of cancer in 2018 and that this number could double by 2040.

In Brazil, the Ministry of Health estimated around 625,000 new cases of cancer per year from 2020 to 2022^(2)^. The highest incidence is that of non-melanoma skin cancer, with approximately 177,000 cases, followed by breast and prostate (66,000 each), colon and rectum (41,000), lung (30,000), and stomach cancer (21,000).

A plan was devised to curb the growth of new cases of neoplasia, implementing prevention measures, early diagnosis, treatment, surveillance, and palliative care^(1)^.

However, in 2020, the new coronavirus (COVID-19) pandemic negatively impacted the diagnosis and treatment of cancer, and the result of not seeking early treatment will be revealed over the next few years^(3)^.

In September 2021, the Federal Speech-Language-Hearing (SLH) Council published the regulations for the work of SLH pathologists in palliative care^(4)^. It should aim at qualifying and rehabilitating SLH aspects from diagnosis to terminality. Thus, it seeks to avoid and/or delay the patient’s loss of functioning and help them maintain safe and enjoyable independence for as long as possible^(4)^.

However, the patient tends to remain relatively stable for a variable time if cancer is diagnosed in the early stages of its natural course and disease-modifying treatments are started (e.g., chemotherapy, radiotherapy, and surgery, either alone or in combination). This is because complications may occur over time, possibly resulting in a sudden decline in the patient's functioning – although this loss is reversible when the complications are resolved. Therefore, at a certain point, there is a decline in functioning due to the progression of the disease, which results in the death of the patient^(5)^.

Therefore, the disease process can trigger dysphagia, even if it is not head and neck cancer, due to medications, modifying treatment, or loss of muscle mass (cachexia and anorexia syndrome). This affects the patient's quality of life^(6,7)^, as food provides not only energy but also the pleasure of eating and having emotional memories, which are unchanged despite the disease^(8)^.

Palliative care aims to promote the quality of life of patients and their families. Hence, it is crucial to evaluate and monitor patients with dysphagia to provide adequate management and a safe diet and improve their well-being^(4,7)^. Nonetheless, some palliative care services do not have SLH pathologists on their teams, even though international^(9)^ and national studies^(10)^ highlight the importance of this professional in managing dysphagia and reducing its impact on the patient's life. However, these studies that address the impact of dysphagia on quality of life did not correlate with the patient's functional status, but rather with the cancer site, sex, lifestyle habits, education, and socioeconomic status^(10)^.

Thus, understanding that the disease process and loss of functioning can impact the patients, this study hypothesized that if dysphagia impacts their quality of life, this clinical condition must also be related to functional performance. Therefore, it is important to evaluate the impact of dysphagia and its relationship with the functional status of cancer patients in palliative care since in each phase of the illness the subject manifests different particularities. Thus, this research aimed to correlate the quality of life in dysphagia with the performance of cancer patients in palliative care, contributing to SLH practice and the patients’ well-being.

## METHOD

This research was approved by the Institution's Research Ethics Committee, under evaluation report no. 6.211.822. Data were collected from November 2022 to August 2023. This analytical, observational, cross-sectional study was carried out in the outpatient clinic and oncology ward of a Hospital in Rio Grande do Norte, Brazil, with a convenience sample of 39 patients aged 45 to 88 years (mean of 65.3 years; ± 10.89), of which 24 (61.5%) were women and 15 (38.5%) were men. Also, 29 (74.4%) patients were from the outpatient clinic, and only 10 (25.6%) were from the ward.

The inclusion criteria were oncology patients aged 18 years or older who answered positively to the question, “Do you have difficulty or problems swallowing?”. All participants signed an informed consent form. Then, information was collected from the medical records regarding age, sex, cancer type and site, feeding route, and food consistency. Only inpatients had data on their diet recorded by the nutrition team. Those who were in the outpatient clinic were asked about how their diet was prepared at home and what its consistency was. To categorize the various nomenclatures described by the patients and team, oral (OR) diets were grouped as “OR without adaptation” (diets described without adaptation of the consistency), “OR with adaptation” (liquidized, liquid, and pureed food), and “OR – Alternative feeding route” (any means of feeding other than orally).

Patients with head and neck cancer were excluded from the study, as the number of patients with this profile is small in the service where the study was conducted, so these could be confounding factors. Patients who were in the active process of dying, in a state of drowsiness, in extreme pain, or in systemic instability at the time of the interview and who did not complete the instruments completely were also excluded.

The perception of the impact of dysphagia on the patients’ quality of life was assessed with the M. D. Anderson Dysphagia Inventory (MDADI) (Annex A), translated and validated for Brazilian Portuguese^(11,12)^, and designed for patients treated for head and neck cancer.

Although this is not the target audience of this research, it is the instrument that most closely matches our object of study. It has 20 questions, divided into four subscales: global (1 item), emotional (6 items), functional (5 items), and physical (8 items). Except for the global subscale, which is scored individually, the total score for the other categories is obtained by adding the points for each category, calculating its mean, and multiplying it by 20. The proposed levels of swallowing limitation are 0-20: profound limitation; 21-40: severe limitation; 41-60: moderate limitation; 61-80: medium limitation; 81-100: minimal limitation^(13)^. During the initial application to hospitalized patients, it was observed that they had difficulty reading and responding to the questionnaire in writing. The evaluator then had to read the sentences and the five possible answers: “Strongly agree”, “Agree”, “No opinion”, “Disagree”, and “Strongly disagree”.

The patients were previously trained to help them understand the questionnaire, by giving two examples that are not in it: “I have difficulty climbing stairs” and “I would like the window to be opened”, then giving them the five answer options. However, it was necessary to adapt the way the answers were given. The opposite ones were given first: “Agree”, “No opinion”, and “Disagree”. If the patient said they agreed, the second confirmation stage was carried out with two answer options: “Strongly agree” or “Agree”; the same was carried out if the patient said they disagreed.

The Palliative Performance Scale (PPS) was chosen and applied to assess the degree of functioning on the same day as the other assessments. The first version of the scale was published in 1996^(14)^, but this study used the version proposed by the Victoria Hospice Society^(15)^, translated into Brazilian Portuguese (Annex B). It scores from 100 to 0, with 10-point decrements, in which 100 indicates a healthy individual, and 0 indicates death. The evaluator selects which item the patient fits into according to their current performance. The patient is then evaluated for ambulation, activity and evidence of disease, self-care, intake, and consciousness level.

Data were descriptively analyzed by means of absolute and relative distribution in the case of categorical variables and by calculating measures of mean central tendency and standard deviation. The instrument scores were correlated with Pearson's correlation tests after verifying the normality of the dependent variables with the Kolmogorov Smirnov test, at the 5% significance level.

## RESULTS

The diagnosis described only the primary tumor sites and that the metastasis had not reached the head or neck. Six patients had pancreatic neoplasia (15.4%), followed by stomach cancer (n = 4; 10.3%). The other sites are listed in Table 1. Patients with proximal esophageal cancer were excluded from the sample because the tumor in the region of the upper esophageal sphincter is directly related to oropharyngeal dysphagia, and its radiotherapy and surgical treatment alter the structures involved in swallowing biodynamics^(12,16)^. Thus, only patients with a distal tumor remained in the study.

**Table 1:** Characteristics of tumor sites. Natal, RN, Brazil. 2023

	n	%
Pancreatic cancer	6	15.4
Stomach cancer	4	10.3
Colon cancer	2	5.1
Esophageal cancer	2	5,1
Liver cancer	2	5.1
Breast cancer	2	5.1
Prostate cancer	2	5.1
Lung cancer	2	5.1
Rectosigmoid cancer	2	5.1
Chronic lymphocytic leukemia	2	5.1
Cancer of the ampulla of Vater	1	2.6
Peritoneal cancer	1	2.6
Duodenal cancer	1	2.6
Rectal cancer	1	2.6
Kidney cancer	1	2.6
Bile duct cancer	1	2.6
Myeloproliferative disease	1	2.6
Chronic myeloproliferative disease	1	2.6
Non-Hodgkin’s lymphoma	1	2.6
Diffuse non-Hodgkin’s lymphoma	1	2.6
Primary myelofibrosis	1	2.6
Multiple myeloma	1	2.6
Smoldering myeloma	1	2.6
Total	39	100.0

Caption: CA = Cancer; n= number of participants

Most patients were fed orally without adaptation (n = 24; 61.5%), followed by orally with adaptation (n = 13; 33.3%). Only two of these patients reported using an alternative feeding route, one using a gastrostomy and liquid diet, and the other a nasoenteral tube and liquid comfort diet. Only one patient was not fed orally because they had a jejunostomy (2.6%). Only one patient did not fill in this information and had the data missing (2.6%). The SLH team had not evaluated any of the patients before they were interviewed in the ward. The participants in the outpatient clinic denied having had or sought SLH assistance for the complaint of swallowing difficulties.

The PPS mean score was 66.1 points (±15.1), and the MDADI mean result was 61.9 points (±12.6) (Table 2).

**Table 2:** Description of the functional performance scale and dysphagia quality of life questionnaire scores. Natal, RN, Brazil. 2023

	n	Minimum	Maximum	Mean	SD
PPS	39	40.0	100.0	66.1	15.1
MDADI – total*	39	35.3	81.7	61.9	12.6
MDADI – global	39	20.0	100.0	50.2	23.7
MDADI – emotional	39	30.0	90.0	62.7	16.2
MDADI – functional	39	32.0	96.0	67.3	14.6
MDADI – physical	39	32.5	80.0	55.6	13.2

* MDADI total = sum of the MDADI domains

Caption: PPS = Palliative Performance Scale; MDADI = M. D. Anderson Dysphagia Inventory; SD = standard deviation; n= number of participants

The analysis with the entire sample found that PPS was moderately negatively correlated with age (p = 0.025) (Table 3), indicating that as age increases, the PPS score decreases. However, the division between outpatient and ward patients found that MDADI was moderately positively correlated with PPS (r = 0.397; p = 0.033) (Table 4). The division between sexes (Table 5) found that age correlated with PPS only in the group of women (r = -0.566; p = 0.004), indicating that as age increases, functional performance decreases in the female group. Likewise, the MDADI total and emotional scores also decreased, with respectively (r = -0.435; p = 0.034) and (r = -0.491; p = 0.015). Moreover, women had a decrease in the emotional and physical domains according to the decrease in the PPS result (r = 0.408; p = 0.048) and (r = 0.424; p = 0.039), respectively. Only in the male group increasing age was positively correlated with the MDADI global score (r = 0.540; p = 0.038).

**Table 3:** Correlation between MDADI total and domain scores, PPS, and age in the whole sample. Natal, RN, Brazil. 2023

		PPS	MDADI total*	MDADI global	MDADI emotional	MDADI functional	MDADI physical
AGE	r	-0.358**	-0.264	-0.069	-0.294	-0.158	-0.217
	p-value	**0.025**	0.104	0.675	0.069	0.336	0.185
	n	39	39	39	39	39	39
PPS	r	1	0.146	0.142	0.186	-0.061	0.257
	p-value		0.376	0.390	0.256	0.711	0.114
	n	39	39	39	39	39	39

* MDADI total = sum of the MDADI domains;

** significant values (p < 0.05)

Caption: PPS = Palliative Performance Scale; MDADI = M. D. Anderson Dysphagia Inventory; r = Pearson correlation; n= number of participants

**Table 4:** Correlation between MDADI total score and PPS in the entire sample, separated by collection locations. Natal, RN, Brazil. 2023

LOCATION			PPS
OUTPATIENT CLINIC	MDADI total*	r	0.397**
		p-value	**0.033**
		n	29
WARD		r	-0.377
		p-value	0.282
		n	10

* MDADI total = sum of the MDADI domains;

** significant values (p < 0.05)

Caption: PPS = Palliative Performance Scale; MDADI = M. D. Anderson Dysphagia Inventory; r = Pearson correlation; n= number of participants

**Table 5:** Correlation between MDADI total and domain scores, PPS, and age, divided by sex. Natal, RN, Brazil. 2023

			PPS	MDADI total*	MDADI global	MDADI emotional	MDADI functional	MDADI physical
Males	AGE	r	0.003	0.081	0.540**	0.049	0.034	0.140
		p-value	0.992	0.774	**0.038**	0.862	0.905	0.620
		n	15	15	15	15	15	15
	PPS	r	1	-0.152	0.102	-0.062	-0.320	0.020
		p-value		0.590	0.718	0.826	0.245	0.943
		n	15	15	15	15	15	15
Females	AGE	r	-0.566**	-0.435**	-0.316	-0.491*	-0.259	-0.363
		p-value	**0.004**	**0.034**	0.133	**0.015**	0.222	0.082
		n	24	24	24	24	24	24
	PPS	r	1	0.381	0.163	0.408**	0.146	0.424**
		p-value		0.066	0.447	**0.048**	0.495	**0.039**
		n	24	24	24	24	24	24

* MDADI total = sum of the MDADI domains;

** significant values (p < 0.05)

Caption: PPS = Palliative Performance Scale; MDADI = M. D. Anderson Dysphagia Inventory; r = Pearson correlation; n= number of participants

## DISCUSSION

The study revealed that PPS was moderately positively correlated with MDADI in outpatients, indicating that the perception of the impact of dysphagia increases as functioning decreases^(17)^. Even though the literature reported the influence of dysphagia on quality of life in patients without head and neck cancer^(10,18)^, These studies did not correlate such data with functional performance.

This correlation can be attributed to frailty, since the decreased functional capacity affects basic activities of daily living, resulting in physical, emotional, and cognitive fatigue in frail patients^(19)^. This also justifies the correlation that differed between sexes, as women had lower scores in the emotional and physical domains as PPS decreased. Thus, feeding difficulties may have remained constant, but due to frailty, patients began to perceive a greater impact of dysphagia on quality of life, identified by the MDADI, which is sensitive to health-related emotional and psychological aspects^(11)^.

This study did not assess swallowing; rather, it investigated only the presence of swallowing complaints. Therefore, it cannot be stated that the correlation between PPS and MDADI scores is due to greater severity of dysphagia in patients with lower functioning. However, decreased muscle mass, sarcopenia, oncological disease^(20,21)^ and aging^(22)^ (the mean age in this research was 65.3 years) may be result in this correlation, as it impacts both general functioning and, specifically, mastication and swallowing^(23)^.

This study focused on the influence of dysphagia on quality of life and functioning. Previous research on the subject that did not consider functioning raises the question of whether the data refer to patients with high functioning and therefore had a minor decrease in quality of life or whether dysphagia maintains a reduced impact on quality of life even in an advanced disease stage^(10,18)^. This study found that patients with an intermediate PPS score complained of dysphagia and had a medium impact on their quality of life. Similarly, despite not finding a statistically significant difference, a study showed that patients with more advanced tumors tended to have a lower MDADI score^(11)^.

At the onset of the disease, the literature recommends offering enteral nutritional treatment to optimize the response to oncological therapy and improve the quality of life^(24)^. This measure is mostly used in palliative care patients with head and neck cancer or upper gastrointestinal tumors^(25)^. As seen in the public of this study, it is not uncommon to find patients with an alternative feeding route associated with the oral route to provide a comfort diet or transition to the oral route^(26,27)^. However, as the disease progresses, it is necessary to undergo a new assessment involving a multidisciplinary team, family members, and patients and thus establish what the possibilities and priorities are at the new moment^(24)^.

The number of participants in this study who reported complaints of dysphagia and were on oral feeding reached 94.8% of the sample. It is not uncommon to find a high percentage of patients in palliative care on oral feeding^(10,27)^. This finding is justified by the PPS result, which was 66.1 points (±15.15), indicating that this population has a normal or reduced intake. The SLH pathologist of the palliative care team may decide to maintain the patient's oral diet to promote their satisfaction and quality of life. However, it is important to recognize that, even with consistency adjustments and specific techniques, no diet is completely risk-free^(28)^. Furthermore, the fact that these patients do not have head and neck cancer also has an influence, since tumors in these regions cause pain and changes in swallowing structures, and their treatment also leads to swallowing impairments, making oral feeding more difficult^(16)^, resulting in further swallowing limitations.

The tumor sites found in this study did not follow the ranking of cancer types predicted for the three-year 2023-2025 period, which are breast cancer and prostate cancer^(29)^. The most frequent diagnoses in this study were pancreatic cancer, which ranks 14^th^, and stomach cancer, which ranks 5^th^ among the most frequent cancers in Brazil, excluding non-melanoma skin tumors^(29)^. Patients with pancreatic tumors have symptoms such as fatigue, weight loss, and abdominal pain, and patients with stomach cancer have fatigue, nausea, vomiting, and loss of appetite – besides the treatment, whose effects potentiate the stress experienced by the patient, impacting their quality of life and reducing their functioning^(30,31)^.

The moderate negative correlation between age and PPS without dividing the sample into male and female groups demonstrates decreased functional performance with increasing age in these patients^(17)^. Older people often have other comorbidities^(32,33)^ which combined impact their functioning, and those with neoplasms may also be fatigued and frail^(19)^. Furthermore, the analysis between sexes found that age moderately negatively correlated with the MDADI total and emotional scores only in women. In another study, women also demonstrated a greater impact of dysphagia on quality of life^(11)^.

In the group of men, age moderately positively correlated with the MDADI global score. However, the literature reports that women had lower scores than men^(11)^, not that they have a better quality of life with dysphagia as they get older. No data were found in the literature to support this finding.

This study had limitations regarding the few patients recruited in the ward compared to the outpatient clinic. Another limitation of the study is that the researcher had to dictate the sentences and answer options due to the patients’ difficulty in reading and answering the questionnaire. These variables may have influenced the study and should be considered in future research.

## CONCLUSION

The study demonstrated that functional performance was moderately correlated with the impact of dysphagia on the quality of life in cancer patients in the palliative care outpatient clinic.

## Annex A. *M. D. Anderson Dysphagia Inventory* (MDADI)

Name:__________________________________ MRN:__________________ Date:________________ () TEST ()RETEST

This questionnaire asks for your views about your swallowing ability. This

information will help us understand how you feel about swallowing.

The following statements have been made by people who have problems with their

swallowing. Some of the statements may apply to you.

Please read each statement and circle the response which best reflects your

experience in the past week.

My swallowing ability limits my day-to-day activities.

() Strongly Agree () Agree () No Opinion () Disagree () Strongly Disagree

E2. I am embarrassed by my eating habits.

() Strongly Agree () Agree () No Opinion () Disagree () Strongly Disagree

F1. People have difficulty cooking for me.

() Strongly Agree () Agree () No Opinion () Disagree () Strongly Disagree

P2. Swallowing is more difficult at the end of the day.

() Strongly Agree () Agree () No Opinion () Disagree () Strongly Disagree

E7. I do not feel self-conscious when I eat.

() Strongly Agree () Agree () No Opinion () Disagree () Strongly Disagree

E4. I am upset by my swallowing problem.

() Strongly Agree () Agree () No Opinion () Disagree () Strongly Disagree

P6. Swallowing takes great effort.

() Strongly Agree () Agree () No Opinion () Disagree () Strongly Disagree

E5. I do not go out because of my swallowing problem.

() Strongly Agree () Agree () No Opinion () Disagree () Strongly Disagree

F5. My swallowing difficulty has caused me to lose income.

() Strongly Agree () Agree () No Opinion () Disagree () Strongly Disagree

P7. It takes me longer to eat because of my swallowing problem.

() Strongly Agree () Agree () No Opinion () Disagree () Strongly Disagree

P3. People ask me, “Why can’t you eat that?”

() Strongly Agree () Agree () No Opinion () Disagree () Strongly Disagree

E3. Other people are irritated by my eating problem.

() Strongly Agree () Agree () No Opinion () Disagree () Strongly Disagree

P8. I cough when I try to drink liquids.

() Strongly Agree () Agree () No Opinion () Disagree () Strongly Disagree

F3. My swallowing problems limit my social and personal life.

() Strongly Agree () Agree () No Opinion () Disagree () Strongly Disagree

F2. I feel free to go out to eat with my friends, neighbors, and relatives.

() Strongly Agree () Agree () No Opinion () Disagree () Strongly Disagree

P5. I limit my food intake because of my swallowing difficulty.

() Strongly Agree () Agree () No Opinion () Disagree () Strongly Disagree

P1. I cannot maintain my weight because of my swallowing problem.

() Strongly Agree () Agree () No Opinion () Disagree () Strongly Disagree

E6. I have low self-esteem because of my swallowing problem.

() Strongly Agree () Agree () No Opinion () Disagree () Strongly Disagree

P4. I feel that I am swallowing a huge amount of food.

() Strongly Agree () Agree () No Opinion () Disagree () Strongly Disagree

F4. I feel excluded because of my eating habits.

() Strongly Agree () Agree () No Opinion () Disagree () Strongly Disagree

Thank you for completing this questionnaire!

## Annex B. *Palliative Performance Scale* (PPS) – (Victoria Hospice Society, 2009)

Portuguese Brazilian translation of Palliative Performance Scale (PPS version 2)

**Table:**

PPS Level	Ambulation	Activity & Evidence of Disease	Self-Care	Intake	Conscious Level
PPS 100%	Full	Normal activity & work No evidence of disease	Full	Normal	Full
PPS 90%	Full	Normal activity & work Some evidence of disease	Full	Normal	Full
PPS 80%	Full	Normal activity with Effort Some evidence of disease	Full	Normal or reduced	Full
PPS 70%	Reduced	Unable Normal Job/Work Significant disease	Full	Normal or reduced	Full
PPS 60%	Reduced	Unable hobby/house work Significant disease	Occasional assistance necessary	Normal or reduced	Full or Confusion
PPS 50%	Mainly Sit/Lie	Unable to do any work Extensive disease	Considerable assistance required	Normal or reduced	Full or Confusion
PPS 40%	Mainly in Bed	Unable to do most activity Extensive disease	Mainly assistance	Normal or reduced	Full or Drowsy +/- Confusion
PPS 30%	Totally Bed Bound	Unable to do any activity Extensive disease	Total care	Normal or reduced	Full or Drowsy +/- Confusion
PPS 20%	Totally Bed Bound	Unable to do any activity Extensive disease	Total care	Minimal to sips	Full or Drowsy +/- Confusion
PPS 10%	Totally Bed Bound	Unable to do any activity Extensive disease	Total care	Mouth care only	Drowsy or Coma +/- Confusion
PPS 0%	Death	-	-	-	-
