# Supplementary material for: Quality of life in dysphagia and functional performance of cancer patients in palliative care
Source: Codas. 2024 Sep 9;36(5):e20230266. doi: 10.1590/2317-1782/20242023266en (PMC11404836; doi:10.1590/2317-1782/20242023266en)
Supplement: Translation

# Translation: Qualidade de vida em disfagia e performance funcional de pacientes oncológicos em cuidados paliativos

**DOI:** 10.1590/2317-1782/20242023266pt

**Authors:** Laressa Cardoso Barbosa, Leandro de Araújo Pernambuco, Hipólito Magalhães

**Affiliations:** 1 Programa de Pós-graduação em Fonoaudiologia, Universidade Federal do Rio Grande do Norte – UFRN - Natal (RN), Brasil.; 2 Departamento de Fonoaudiologia, Universidade Federal de Pernambuco – UFPE - Recife (PE), Brasil.; 3 Departamento de Fonoaudiologia, Universidade Federal do Rio Grande do Norte – UFRN - Natal (RN), Brasil.

**Keywords:** Transtornos de Deglutição, Qualidade de Vida, Cuidados Paliativos, Oncologia, Neoplasias, Estado Funcional

## Abstract

**Objetivo:**

Correlacionar a performance funcional e impacto da disfagia na qualidade de vida de pacientes oncológicos em cuidados paliativos.

**Método:**

Estudo transversal e quantitativo realizado no ambulatório e enfermaria de oncologia de um hospital universitário. Os critérios de inclusão exigiram que os pacientes respondessem positivamente à pergunta: “você tem dificuldade ou problema para engolir?”. Foram excluídos os pacientes que tivessem diagnóstico de câncer de cabeça e pescoço, incapacidade de responder questionários devido a estarem em processo ativo de morte, estado de sonolência, dor extrema e instabilidade sistêmica, bem como os instrumentos de coleta que não foram concluídos. Foram aplicados dois instrumentos em suas versões para o português brasileiro: a Palliative Performance Scale (PPS) e M. D. Anderson Dysphagia Inventory (MDADI). A análise das variáveis foi realizada com base na estatística descritiva e inferencial, por meio da correlação de Pearson, em nível de significância de 5%.

**Resultados:**

A amostra foi composta por 39 participantes, com média de 65,3 anos, dos quais 24 (61,5%) eram mulheres. As localizações mais frequentes de neoplasia foram: pâncreas e estômago. O resultado da PPS indicou que a média dos pacientes apresentou deambulação reduzida, incapacidade para trabalhar, porém com independência no autocuidado, nível de ingesta e consciência completos e o MDADI obteve grau médio de limitação. Pacientes ambulatoriais apresentaram correlação moderada entre o resultado do MDADI e nível de funcionalidade pela PPS.

**Conclusão:**

Pacientes oncológicos do ambulatório de cuidados paliativos apresentaram correlação entre performance funcional e o impacto da disfagia na qualidade de vida.

## INTRODUÇÃO

O câncer é um dos grandes problemas de saúde que atinge todas as populações, observado de forma mais prevalente em países de média e baixa renda devido a limites de recursos investidos na saúde, seja na prevenção ou tratamento^(1)^. Dentre os tipos mais prevalentes, encontra-se o câncer de pulmão (11,6% de todos os casos), seguido pelo câncer de mama em mulheres (11,6%) e em terceiro lugar o câncer de colorretal (10,2%). A OMS estimou que, em 2018, 18,1 milhões de pessoas em todo o mundo tiveram alguma doença oncológica e que em 2040, esse número poderá dobrar.

No Brasil, segundo o Ministério da Saúde, para cada ano do triênio 2020-2022 a estimativa era que ocorreriam cerca de 625 mil casos novos de câncer^(2)^. Em primeiro lugar no número de incidência, está o câncer de pele não melanoma com aproximadamente 177 mil casos, seguido pelos cânceres de mama e próstata (66 mil cada), cólon e reto (41 mil), pulmão (30 mil), e estômago (21 mil).

Com intuito de frear o crescimento dos novos casos de neoplasias, um planejamento foi elaborado para implementar medidas de prevenção, diagnóstico precoce, tratamento, vigilância e cuidados paliativos^(1)^.

Todavia, em 2020, a pandemia do novo Coronavírus (COVID-19) impactou negativamente o diagnóstico e o tratamento do câncer, cujo resultado da não busca por um tratamento precoce será revelado ao longo dos próximos anos^(3)^.

Em setembro de 2021, o Conselho Federal de Fonoaudiologia publicou a regulamentação da atuação do fonoaudiólogo nos Cuidados Paliativos^(4)^. A atuação deste deve visar a habilitação e reabilitação dos aspectos fonoaudiológicos desde o diagnóstico até a terminalidade. E, dessa forma, busca evitar e/ou retardar a perda de funcionalidade do paciente e promover a manutenção de sua independência de forma segura e prazerosa pelo maior tempo possível^(4)^.

Porém, se tratando do percurso natural da doença oncológica, se o diagnóstico é realizado na fase inicial e logo os tratamentos modificadores de doença são iniciados como quimioterapia, radioterapia e cirurgia, sejam de forma isolada ou combinadas, o paciente tende a se manter relativamente estável por um tempo variável. Isto porque podem acontecer intercorrências ao longo do tempo e com isso ter uma queda abrupta da funcionalidade do paciente, mas esta perda é reversível com a resolubilidade da intercorrência. Então, em um determinado momento ocorre o declínio da funcionalidade devido a progressão da doença, que resulta na morte do paciente^(5)^.

Portanto, o processo de adoecimento pode desencadear a disfagia, ainda que o câncer não seja de cabeça e pescoço, seja por causa de medicações, tratamento modificador, pela perda de massa muscular (síndrome de caquexia e anorexia), o que afeta a qualidade de vida do paciente^(6,7)^, pois, a alimentação não está relacionada apenas a ganhos energéticos, mas também envolve o prazer de se alimentar e memórias afetivas que apesar do adoecimento, esses valores não são alterados^(8)^.

O objetivo dos Cuidados Paliativos é promover a qualidade de vida dos pacientes e familiares, o que torna crucial avaliar e acompanhar pacientes com disfagia para proporcionar um manejo adequado e uma dieta segura, bem como melhora no bem-estar^(4,7)^. Apesar disso, alguns serviços de cuidados paliativos não contam com fonoaudiólogos em suas equipes, embora estudos internacionais^(9)^ e nacionais^(10)^ destaquem a importância desse profissional no manejo da disfagia e na redução de seus impactos na vida do paciente. Todavia, esses estudos que abordam o impacto da disfagia na qualidade de vida não correlacionaram com o status funcional do paciente, mas sim com a localização do câncer, sexo, hábitos de vida, escolaridade e status socioeconômicos^(10)^.

Portanto, ao compreender que o processo de adoecimento e a perda de funcionalidade podem impactar nos sujeitos adoecidos, a hipótese desse estudo é que se a disfagia impacta em sua qualidade de vida, essa condição clínica também deve estar relacionada com a performance funcional. Por isso faz-se importante avaliar o impacto da disfagia e sua relação com o status funcional de pacientes oncológicos em cuidados paliativos, pois em cada fase do adoecimento o sujeito manifesta particularidades distintas. Desta forma, com o intuito de colaborar com a prática dos fonoaudiólogos e o bem-estar do paciente, esta pesquisa visa correlacionar a qualidade de vida em disfagia com a performance dos pacientes oncológicos em cuidados paliativos.

## MÉTODO

Esta pesquisa foi aprovada pelo Comitê de Ética em Pesquisa da Instituição, sob o parecer de nº 6.211.822. O período de coleta foi de novembro de 2022 a agosto de 2023. Trata-se de um estudo transversal, observacional e analítico, realizado em ambulatório e enfermaria de oncologia de um Hospital do Rio Grande do Norte, constituído de amostra por conveniência, com 39 pacientes com idades entre 45 e 88 anos (média de 65,3 anos; ±10,89), dos quais 24 (61,5%) eram mulheres e 15 (38,5%) eram homens. Quanto à origem dos participantes 29 (74,4%) foram do ambulatório e apenas 10 (25,6%) da enfermaria.

Os critérios de inclusão foram ser paciente oncológico com idade igual ou superior a 18 anos e ter respondido positivamente à pergunta “Você tem dificuldade ou problema para engolir?”. Todos os participantes assinaram o Termo de Consentimento Livre e Esclarecido. Após isso, foram coletadas informações nos prontuários referentes à idade, gênero, tipo e localização do câncer e via de alimentação bem como consistência da mesma. Apenas pacientes internados tinham dados sobre dieta registrada pela equipe de Nutrição. Aos que eram do ambulatório, foi feito o questionamento sobre como era o preparo e consistência da sua dieta em casa. A fim de categorizar as diversas nomenclaturas descritas pelos pacientes e equipe foram agrupadas as dietas por Via Oral (VO) como “VO sem adaptação” são dietas descritas sem adaptação da consistência, “VO com adaptação” que foram descritas como liquidificada, líquida e pastosa e “VA – Via alternativa de alimentação” qualquer outro meio de alimentação que não fosse por via oral.

Foram excluídos da pesquisa pacientes com câncer de cabeça e pescoço, pois no serviço em que foi realizada a pesquisa o número de pacientes deste perfil é reduzido, então esses poderiam ser fatores de confundimento. Ainda foram excluídos, pacientes que estavam em processo ativo de morte, estado de sonolência, dor extrema e instabilidade sistêmica no momento da entrevista e que não preencheram completamente os instrumentos.

Para avaliar a percepção do impacto da disfagia na qualidade de vida dos pacientes, foi aplicado o Questionário de Disfagia M. D. Anderson (MDADI) (Anexo A), que foi traduzido e validado para o português brasileiro^(11,12)^ e idealizado para pacientes que receberam o tratamento para câncer de cabeça e pescoço.

Embora esse não fosse para o público-alvo desta pesquisa, é o instrumento que mais se aproxima do nosso objeto de estudo. Possui 20 questões, divididas entre quatro categorias: global (1 item), emocional (6 item), funcional (5 item) e física (8 item). Exceto a categoria global, que é pontuada de forma individual, a pontuação total das demais categorias é resultante da soma dos pontos de cada categoria, extração de sua média, que deve ser multiplicada por 20. Os níveis propostos de limitação da deglutição, são: 0-20: limitação profunda; 21-40: limitação severa; 41-60: limitação moderada; 61-80: limitação média; 81-100: limitação mínima^(13)^. Durante o período inicial de aplicação com os pacientes internados, foi observado a dificuldade destes em ler e responder de forma escrita o questionário. Então foi necessário o avaliador fazer a leitura das sentenças e das 5 possíveis respostas “concordo totalmente”, “concordo”, “sem opinião”, “discordo” e “discordo totalmente”.

Para maior compreensão do questionário foi realizado um treino prévio com os pacientes. Nesse treino foram oferecidos dois exemplos que não estão no questionário “Eu tenho dificuldade em subir escadas” e “Eu gostaria que abrissem a janela” e em seguida foram são apresentadas as cinco possibilidades de resposta. Porém, foi necessário adaptar a forma de oferecer as respostas. Primeiro, foram apresentadas as opções opostas: “concordo,” “sem opinião” e “discordo.” Se o paciente dissesse que concordava, foi realizada a segunda etapa de confirmação, na qual se ofereciam duas respostas: “concordo totalmente” ou “concordo.” Da mesma forma, isso ocorria se o paciente mencionasse que discordava.

Para avaliar o grau de funcionalidade foi escolhida a *Palliative Performance Scale* (PPS) que foi aplicada no mesmo dia das demais avaliações. A primeira versão da escala foi publicada em 1996^(14)^, porém neste estudo foi usado a versão proposta pela *Victoria Hospice Society* ^(15)^, traduzida para o português brasileiro (Anexo B). Esta escala pontua de 100 a 0, com decréscimos de 10 pontos, em que 100 indica um indivíduo saudável e 0 representa a morte. O avaliador seleciona a qual item o paciente se enquadra segundo sua performance atual. Então o paciente é avaliado quanto a deambulação, atividade e evidência da doença, autocuidado, ingesta e nível de consciência.

A análise dos dados foi feita de modo descritivo por meio de distribuição absoluta e relativa no caso das variáveis categóricas e pelo cálculo de medidas de tendência central média e desvio padrão. A correlação entre os escores dos instrumentos aplicados foi realizada por meio do teste de correlação de *Pearson* após ter sido verificada a normalidade das variáveis dependentes, por meio do teste de *Kolmogorov Smirnov*, em nível de significância de 5%.

## RESULTADOS

Quanto ao diagnóstico, foram descritos apenas os locais de tumores primários e que a metástase não alcançasse a região de cabeça e pescoço. A neoplasia de pâncreas ocorreu em seis pacientes (15,4%), seguido do diagnóstico de câncer de estômago (n=4; 10,3%). As demais localizações estão listadas na Tabela 1. Os pacientes que apresentaram câncer de esôfago proximal foram excluídos da amostra pois o tumor na região do esfíncter esofágico superior está diretamente relacionado com a disfagia orofaríngea, bem como seu tratamento radioterápico e cirúrgico alteram as estruturas envolvidas na biodinâmica da deglutição^(12,16)^. Restando apenas pacientes com o tumor em região distal.

**Tabela 1:** Características da localização dos tumores. Natal, RN. 2023

	n	%
CA de pâncreas	6	15,4
CA de estômago	4	10,3
CA de cólon	2	5,1
CA de esôfago	2	5,1
CA de fígado	2	5,1
CA de mama	2	5,1
CA de próstata	2	5,1
CA de pulmão	2	5,1
CA de retossigmóide	2	5,1
Leucemia linfocítica crônica	2	5,1
CA de ampola de vater	1	2,6
CA de peritônio	1	2,6
CA de duodeno	1	2,6
CA de reto	1	2,6
CA de rim	1	2,6
CA de vias biliares	1	2,6
Doença mieloproliferativa	1	2,6
Doença mieloproliferativa crônica	1	2,6
Linfoma não Hodgkin	1	2,6
Linfoma não Hodgkin difuso	1	2,6
Mielofibrose primária	1	2,6
Mieloma múltiplo	1	2,6
Mieloma smoldering	1	2,6
Total	39	100,0

Legenda: CA = Câncer; n= número de participantes

A maior parte dos pacientes se alimentava por via oral sem adaptação (n=24; 61,5%), seguido de via oral com adaptação (n=13; 33,3%). Desses apenas dois pacientes relataram fazer uso de via alternativa de alimentação, um utilizava a gastrostomia e dieta líquida e outro a sonda nasoenteral e dieta líquida de conforto. Apenas um paciente estava sem via oral pois se alimentava por jejunostomia (2,6%). Apenas um paciente não preencheu essa informação e ficou com o dado ausente (2,6%). Até o dia da entrevista com os pacientes da enfermaria nenhum havia sido avaliado pela equipe de Fonoaudiologia. Os participantes do ambulatório negaram ter tido ou buscado uma assistência fonoaudiológica para a queixa de dificuldade de deglutição.

Quanto à escala PPS, houve uma média de 66,1 pontos (±15,1) e o resultado do questionário MDADI obteve média de 61,9 pontos (±12,6) (Tabela 2).

**Tabela 2:** Descrição das pontuações da escala de performance funcional e do questionário de qualidade de vida em disfagia. Natal, RN. 2023

	n	Mínimo	Máximo	Média	DP
PPS	39	40,0	100,0	66,1	15,1
MDADI – total*	39	35,3	81,7	61,9	12,6
MDADI – global	39	20,0	100,0	50,2	23,7
MDADI – emocional	39	30,0	90,0	62,7	16,2
MDADI – funcional	39	32,0	96,0	67,3	14,6
MDADI – física	39	32,5	80,0	55,6	13,2

* MDADI total = somatório dos domínios do MDADI

Legendas: PPS = *Palliative Performance Scale*; MDADI = Questionário de disfagia M. D. Anderson; DP = Desvio padrão; n= número de participantes

Na análise com toda a amostra houve correlação negativa e moderada entre PPS e idade (*p*=0,025) (Tabela 3), o que indica que à medida que a idade aumenta, a pontuação na escala PPS diminui. Entretanto, na divisão entre o público do ambulatório e da enfermaria houve correlação positiva e de grau moderado entre MDADI e PPS (r=0,397; p=0,033) (Tabela 4). Na divisão entre os gêneros (Tabela 5) a correlação entre idade e PPS foi percebida apenas no grupo de mulheres (r=-0,566; p=0,004), indicando que conforme aumenta a idade diminui a performance funcional no grupo feminino, da mesma forma também diminuiu a pontuação total do MDADI e no domínio emocional (r= -0,435; p=0,034) e (r= -0,491; p=0,015), respectivamente. Ainda, mulheres apresentaram diminuição no domínio emocional e físico conforme diminuição do resultado da PPS (r= 0,408; p= 0,048) e (r= 0,424; p= 0,039), respectivamente. Apenas no grupo masculino foi encontrada correlação positiva para o aumento da idade e pontuação no domínio global do MDADI (r= 0,540; p= 0,038).

**Tabela 3:** Correlação entre pontuação MDADI total e seus domínios, PPS e idade em toda amostra. Natal, RN. 2023

		PPS	MDADI total*	MDADIglobal	MDADI mocional	MDADI funcional	MDADI físico
IDADE	r	-0,358**	-0,264	-0,069	-0,294	-0,158	-0,217
	p-valor	**0,025**	0,104	0,675	0,069	0,336	0,185
	n	39	39	39	39	39	39
PPS	r	1	0,146	0,142	0,186	-0,061	0,257
	p-valor		0,376	0,390	0,256	0,711	0,114
	n	39	39	39	39	39	39

* MDADI total = somatório dos domínios do MDADI;

** Valores significativos (p<0,05)

Legenda: PPS = *Palliative Performance Scale*; MDADI = Questionário de disfagia M. D. Anderson; r = Correlação de Pearson; n= número de participantes

**Tabela 4:** Correlação entre pontuação MDADI total e PPS em toda amostra com separação entre local de coleta. Natal, RN. 2023

LOCAL			PPS
AMBULATÓRIO	MDADI total*	r	0,397**
		p-valor	**0,033**
		n	29
ENFERMARIA		r	-0,377
		p-valor	0,282
		n	10

* MDADI total = somatório dos domínios do MDADI;

** Valores significativos (p<0,05)

Legenda: PPS = *Palliative Performance Scale*; MDADI = Questionário de disfagia M. D. Anderson; r = Correlação de Pearson; n= número de participantes

**Tabela 5:** Correlação entre pontuação MDADI total e seus domínios, PPS e idade com divisão entre os gêneros. Natal, RN. 2023

			PPS	MDADI total*	MDADI global	MDADI emocional	MDADI funcional	MDADI físico
Masc	IDADE	r	0,003	0,081	0,540**	0,049	0,034	0,140
		p-valor	0,992	0,774	**0,038**	0,862	0,905	0,620
		n	15	15	15	15	15	15
	PPS	r	1	-0,152	0,102	-0,062	-0,320	0,020
		p-valor		0,590	0,718	0,826	0,245	0,943
		n	15	15	15	15	15	15
Fem	IDADE	r	-0,566**	-0,435**	-0,316	-0,491*	-0,259	-0,363
		p-valor	**0,004**	**0,034**	0,133	**0,015**	0,222	0,082
		n	24	24	24	24	24	24
	PPS	r	1	0,381	0,163	0,408**	0,146	0,424**
		p-valor		0,066	0,447	**0,048**	0,495	**0,039**
		n	24	24	24	24	24	24

* MDADI total = somatório dos domínios do MDADI;

** Valores significativos (p<0,05)

Legenda: PPS = *Palliative Performance Scale*; MDADI = Questionário de disfagia M. D. Anderson; Masc = Masculino; Fem = Feminino; r = Correlação de Pearson; n= número de participantes

## DISCUSSÃO

O estudo revelou uma correlação positiva moderada entre a PPS e o MDADI em pacientes ambulatoriais, indicando que a percepção do impacto da disfagia aumenta à medida que a funcionalidade diminui^(17)^. Embora a influência da disfagia na qualidade de vida em pacientes sem câncer de cabeça e pescoço seja documentada na literatura^(10,18)^, esses estudos não correlacionaram tais dados com a performance funcional.

Essa correlação pode ser atribuída à fragilidade, uma vez que a diminuição da capacidade funcional afeta as Atividades Básicas de Vida Diária (ABVD), resultando em fadiga física, emocional e cognitiva em pacientes frágeis^(19)^. Isso também justifica a correlação com distinção entre os gêneros, em que as mulheres apresentaram menor pontuação nos domínios emocional e físico à medida que a PPS diminui. Então, a dificuldade na alimentação pode ter permanecido constante, mas devido à fragilidade, os pacientes passaram a perceber um maior impacto da disfagia na qualidade de vida, identificado pelo MDADI, que possui sensibilidade para aspectos emocionais e psicológicos relacionados à saúde^(11)^.

Nesta pesquisa, não foi realizada a avaliação da deglutição; apenas investigou-se a presença de queixa para deglutir. Portanto, não se pode afirmar que a correlação entre os escores da PPS e o MDADI se deve à maior gravidade da disfagia em pacientes com menor funcionalidade. No entanto, a diminuição da massa muscular, sarcopenia, seja pela doença oncológica^(20,21)^ ou devido ao envelhecimento^(22)^, média de idade nesta pesquisa 65,3 anos, pode ser uma das razões dessa correlação, pois impacta tanto a funcionalidade geral quanto às funções específicas de mastigação e deglutição^(23)^.

A influência da disfagia na qualidade de vida e funcionalidade é o foco de interesse deste estudo. Pesquisas anteriores sobre o tema que não consideraram a funcionalidade levantam a questão se os dados se referem a pacientes com alta funcionalidade e por isso apresentaram pequena redução na qualidade de vida, ou apesar de terem uma doença avançada mantém um impacto reduzido da disfagia na qualidade de vida^(10,18)^. Nesta pesquisa, foi observado que pacientes que possuem uma pontuação intermediária na PPS possuem queixa para disfagia e apresentam um grau médio de impacto em sua qualidade de vida. Da mesma forma, apesar de não terem encontrado diferença estatisticamente significativa, uma pesquisa apontou que pacientes com tumores mais avançados tiveram a tendência a apresentarem escore mais baixo no MDADI^(11)^.

No início da doença, é recomendado pela literatura oferecer o tratamento nutricional enteral com o objetivo de otimizar a resposta à terapia oncológica e promover a qualidade de vida^(24)^. Essa medida é mais usada em pacientes em cuidados paliativos com câncer de cabeça e pescoço ou tumores gastrointestinais mais altos^(25)^. Assim como visto no público deste estudo, não é incomum encontrar pacientes com via alternativa de alimentação associada à via oral, com o intuito de proporcionar uma dieta de conforto ou promover a transição para via oral^(26,27)^. Todavia, com o avançar da doença é necessário passar por uma nova avaliação em equipe multiprofissional, familiares e paciente e desta forma estabelecer quais são as possibilidades e prioridades nesse novo momento^(24)^.

O número de participantes nesta pesquisa que relataram queixa de disfagia e estavam em alimentação por via oral atingiu a marca de 94,8% da amostra. Não é incomum encontrar uma alta porcentagem de pacientes em cuidados paliativos com via oral^(10,27)^. Tal achado é justificado pelo resultado da PPS, que foi de 66,1 pontos (±15,15), isto indica que essa população apresenta uma ingestão normal ou reduzida. O fonoaudiólogo que integra a equipe de cuidados paliativos pode decidir manter a alimentação oral do paciente em busca de promover sua satisfação e qualidade de vida. No entanto, é importante reconhecer que, mesmo com ajustes de consistência e técnicas específicas, não existe uma dieta completamente isenta de riscos^(28)^. Além disso, o fato de os pacientes não terem câncer de cabeça e pescoço também influencia, visto que tumores nessas regiões causam dor, modificações nas estruturas da deglutição e seu tratamento também leva a prejuízos na deglutição, tornando mais difícil a alimentação por via oral^(16)^, o que resulta em mais limitações na deglutição.

A localização dos tumores encontrados nesse estudo não seguiu o ranking dos tipos de câncer previsto para o triênio de 2023-2025, que são o câncer de mama e câncer de próstata^(29)^. O diagnóstico mais frequente neste estudo foi o de câncer de pâncreas, o qual ocupa a 14ª posição e de estômago que está em quinto lugar no ranking dos cânceres mais frequente no Brasil, excluindo-se os tumores de pele não melanoma^(29)^. Pacientes com tumores de pâncreas apresentam sintomas como fadiga, perda de peso e dor abdominal, e os com câncer de estômago apresentam fadiga, náusea, vômito, perda de apetite, além disso o próprio tratamento também causa efeitos que potencializam o estresse e geram impacto na qualidade de vida e redução da funcionalidade^(30,31)^.

A correlação negativa e de grau moderado encontrado entre a idade e PPS, sem ter feito a divisão entre o grupo feminino e masculino, demonstra que com o aumento da idade ocorreu a diminuição da performance funcional nestes pacientes^(17)^. Pessoas idosas frequentemente apresentam outras comorbidades^(32,33)^ que combinados geram um impacto na sua funcionalidade, além disso pessoas idosas que apresentam neoplasias podem apresentar fadiga e fragilidade^(19)^. Ainda, na análise entre os gêneros, apenas as mulheres apresentaram correlação negativa e moderada entre idade e escore total da MDADI e no domínio emocional. Em outro estudo as mulheres também demonstraram maior impacto da disfagia na qualidade de vida^(11)^.

Houve correlação positiva de grau moderado no grupo dos homens entre idade e a pontuação no domínio global do MDADI, porém, na literatura é encontrado que as mulheres apresentaram pontuações menores que a dos homens^(11)^, não que eles apresentem uma melhor qualidade de vida em disfagia com o aumento da idade. Não foram encontrados dados na literatura que deem suporte para esse achado.

Esse estudo apresentou limitações quanto ao número reduzido de pacientes coletados na enfermaria em comparação ao ambulatório. Outra limitação do estudo é que devido a dificuldade de leitura para responder ao questionário foi necessário ao pesquisador ditar as sentenças e opções de respostas. Estas variáveis podem ter influenciado o estudo e deverão ser consideradas em pesquisas futuras.

## CONCLUSÃO

O estudo demonstrou que pacientes oncológicos do ambulatório de cuidados paliativos apresentaram correlação moderada entre performance funcional e o impacto da disfagia na qualidade de vida.

## Anexo A. *M. D. Anderson Dysphagia Inventory* (MDADI)

Nome:__________________________________ RGH:__________________ Data:________________ ()TESTE ()RETESTE

Este questionário pergunta sobre sua habilidade de engolir (deglutir). Estas informações irão nos auxiliar a entender como você se sente em relação à sua deglutição. As questões que seguem foram preparadas por pessoas que têm problema com sua deglutição. Alguns dos itens podem ser relevantes para você.

Por favor, leia cada questão e marque a resposta que melhor reflete sua experiência na última semana.

Minha capacidade de deglutição limita minhas atividades diárias

()Concordo totalmente ()Concordo ()Sem opinião ()Discordo ()Discordo totalmente

E2. Eu tenho vergonha dos meus hábitos alimentares

()Concordo totalmente ()Concordo ()Sem opinião ()Discordo ()Discordo totalmente

F1. As pessoas têm dificuldade de cozinhar para mim

()Concordo totalmente ()Concordo ()Sem opinião ()Discordo ()Discordo totalmente

P2. É mais difícil engolir no fim do dia

()Concordo totalmente ()Concordo ()Sem opinião ()Discordo ()Discordo totalmente

E7. Sinto-me inseguro quando me alimento

()Concordo totalmente ()Concordo ()Sem opinião ()Discordo ()Discordo totalmente

E4. Eu estou triste pelo meu problema de deglutição

()Concordo totalmente ()Concordo ()Sem opinião ()Discordo ()Discordo totalmente

P6. Deglutir é um grande esforço

()Concordo totalmente ()Concordo ()Sem opinião ()Discordo ()Discordo totalmente

E5. Deixo de sair de casa por causa do meu problema de deglutição

()Concordo totalmente ()Concordo ()Sem opinião ()Discordo ()Discordo totalmente

F5. Meu problema de deglutição tem me causado perda de rendimentos financeiros

()Concordo totalmente ()Concordo ()Sem opinião ()Discordo ()Discordo totalmente

P7. Eu levo mais tempo pra comer por causa do meu problema de deglutição

()Concordo totalmente ()Concordo ()Sem opinião ()Discordo ()Discordo totalmente

P3. As pessoas me perguntam, “Porque você não pode comer isto?”

()Concordo totalmente ()Concordo ()Sem opinião ()Discordo ()Discordo totalmente

E3. Outras pessoas se irritam por causa do meu problema de deglutição

()Concordo totalmente ()Concordo ()Sem opinião ()Discordo ()Discordo totalmente

P8. Eu tenho tosse quando eu tento beber líquidos

()Concordo totalmente ()Concordo ()Sem opinião ()Discordo ()Discordo totalmente

F3. Meus problemas de deglutição atrapalham minha vida pessoal e social

()Concordo totalmente ()Concordo ()Sem opinião ()Discordo ()Discordo totalmente

F2. Eu me sinto à vontade para sair pra comer com meus amigos, vizinhos e parentes

()Concordo totalmente ()Concordo ()Sem opinião ()Discordo ()Discordo totalmente

P5. Eu limito minha alimentação por causa da minha dificuldade de deglutição

()Concordo totalmente ()Concordo ()Sem opinião ()Discordo ()Discordo totalmente

P1. Perco peso devido ao meu problema de deglutição

()Concordo totalmente ()Concordo ()Sem opinião ()Discordo ()Discordo totalmente

E6. Eu tenho baixa auto-estima por causa do meu problema de deglutição

()Concordo totalmente ()Concordo ()Sem opinião ()Discordo ()Discordo totalmente

P4. Eu sinto que estou conseguindo deglutir uma grande quantidade de alimentos

()Concordo totalmente ()Concordo ()Sem opinião ()Discordo ()Discordo totalmente

F4. Eu me sinto isolado por causa dos meus hábitos de alimentação

()Concordo totalmente ()Concordo ()Sem opinião ()Discordo ()Discordo totalmente

Obrigado por completar este questionário!

## Anexo B. *Palliative Performance Scale* (PPS) – (Victoria Hospice Society, 2009)

A Escala de Desempenho em Cuidados Paliativos versão 2 (EDCP v2)

TRADUÇÃO BRASILEIRA PARA A LÍNGUA PORTUGUESA

(Portuguese Brazilian translation of Palliative Performance Scale (PPS version 2))

**Table:**

PPS	Deambulação	Atividade e evidência da doença	Auto‐cuidado	Ingesta	Nível da Consciência
PPS 100%	Completa	Atividade normal e trabalho; sem evidência de doença	Completo	Normal	Completa
PPS 90%	Completa	Atividade normal e trabalho; alguma evidência de doença	Completo	Normal	Completa
PPS 80%	Completa	Atividade normal com esforço; alguma evidência de doença	Completo	Normal ou reduzida	Completa
PPS 70%	Reduzida	Incapaz para o trabalho; doença significativa	Completo	Normal ou reduzida	Completa
PPS 60%	Reduzida	Incapaz para hobbies/trabalho doméstico; doença significativa	Assistência ocasional	Normal ou reduzida	Completa ou períodos de confusão
PPS 50%	Maior parte de tempo sentado ou deitado	Incapacitado para qualquer trabalho; doença extensa	Assistência considerável	Normal ou reduzida	Completa ou períodos de confusão
PPS 40%	Maior parte do tempo acamado	Incapaz para a maioria das atividades; doença extensa	Assistência quase completa	Normal ou reduzida	Completa ou sonolência +/‐ confusão
PPS 30%	Totalmente acamado	Incapaz para qualquer atividade; doença extensa	Dependência completa	Normal ou Reduzida	Completa ou sonolência +/‐ confusão
PPS 20%	Totalmente acamado	Incapaz para qualquer atividade;doença extensa	Dependência completa	Mínima a pequenos goles	Completa ou sonolência +/- confusão
PPS 10%	Totalmente acamado	Incapaz para qualquer atividade; doença extensa	Dependência completa	Cuidados com a boca	Sonolento ou coma +/- confusão
PPS 0%	Morte	-	-	-	-
